# Motor Imagery Training Improves Interoception and Satisfaction with Performance

**DOI:** 10.3390/medicina61040734

**Published:** 2025-04-16

**Authors:** Chiara Di Tella, Enrica L. Santarcangelo

**Affiliations:** Department of Translational Research and New Technologies in Medicine and Surgery (NTMS), University of Pisa, Via San Zeno, 31, 56127 Pisa, Italy; c.ditella@studenti.unipi.it

**Keywords:** motor imagery, interoceptive sensibility, interoceptive accuracy, performance

## Abstract

*Background and Objectives*: Sport practice, performance satisfaction, and interoception influence physical and mental health. Motor imagery (MI) training improves sensorimotor and cognitive–emotional functions. This study aimed to (a) compare sedentary and artistic gymnastics-practicing young females and (b) evaluate the changes in interoception and performance satisfaction occurring in gymnastics-practicing participants after one month of motor imagery training. *Materials and Methods*: The difference in interoceptive accuracy (IA) and sensibility (IS) between young sedentary females (Control group, C, n = 27) and age-matched females practicing artistic gymnastics (Experimental group, E, n = 27) were studied using the Interoceptive Accuracy Scale (IAS), the Multisensory Assessment of Interoceptive Awareness (MAIA), and Body Perception Questionnaire (BPQ). The capacity for focusing one’s attention on specific tasks (absorption) was assessed by the Tellegen Absorption Scale (TAS). Groups were compared at T0 (before motor imagery training). In group E, the same variables and satisfaction with performance were rated before and after 1 month of motor imagery training. The years of practice and absorption were used as covariates in analyses. *Results*: (a) Group E exhibited significantly higher scores in the MAIA dimensions than group C and similar BPQ and IAS scores; (b) group E’s satisfaction with performance, MAIA, IAS, and BPQ scores increased significantly from T0 to T1. The increase in performance satisfaction became non-significant when using years of practice as the control. The improvement in MAIA dimensions became non-significant when using TAS as the control. *Conclusions*: Despite the limitations as a result of the absence of an objective evaluation of the performance and physiological correlations of mental imagery and interoceptive accuracy, the baseline differences between the two groups confirm that practicing artistic gymnastics improves interoception. The experience undergone by group E of better performance after training is associated with further improvement in interoceptive intermingled pathways and shared relay stations of sensorimotor and interoceptive information. The results are relevant to the setting up of personalized mental training to improve physical and mental health.

## 1. Introduction

In 2019, 44% of the European population practiced physical activities at least once a week. More men (47%) than women (42%) practiced regularly, 65% were young, and 31% were old (https://ec.europa.eu/eurostat/web/main/home, accessed on 9 March 2025). Sports practice improves medical [1,2], neurological [3,4,5,6], and psychological conditions [7,8,9]. Beyond exercise, sports performance satisfaction influences the athletes’ psychological health and sports performance itself [10,11]. In contrast, sports performance dissatisfaction is associated with depression and anxiety [12].

Motor imagery (MI) training improves sports performance [13,14,15].

### 1.1. Motor Imagery

MI is the mental simulation of a motor action without its execution [16]. Actual and imagined movements share similar cortical correlations, except for the inhibition of the primary motor cortex by the supplementary area occurring during imagery. According to the functional equivalence theory [17], the more similar the cortical activities during actual and imagined movement [18] as well as their duration and autonomic correlations [19,20]—the more efficacious the motor imagery. MI is widely used for neurorehabilitation [21,22] and sports [13,14,22,23]. Its efficacy, however, depends on individual imagery abilities regarding the degree of functional equivalence between actual and imagined action; the preferred sensory modality of imagery, i.e., visual or kinesthetic; and the individual’s previous experience of the imagined movement [24]. Actual and imagined movements are influenced by interoception [24].

### 1.2. Interoception

Interoception consists of the perception of the body’s internal state [25], influences physical and mental health [26,27,28], and comprehends sensory and cognitive–emotional components [29], defined as interoceptive accuracy (IA), sensitivity (IS), and awareness (IAW).

IA indicates the ability to detect visceral signals and is measured by self-reported scales [30], behavioral tests such as heartbeat counting [31] or tapping [32], and cortical activities, i.e., the amplitude of heartbeat-evoked cortical potential (HEP) [33]. IS indicates the mode of interpretation of visceral information and is measured by self-reported questionnaires such as the Multisensory Assessment of Interoceptive Awareness (MAIA [34] and the Body Perception Questionnaire (BPQ) [35]. IAW represents the self-reported correspondence between IA and IS.

Interoception is associated with the activity and connectivity of the insula that receives all sensory information, i.e., interoceptive and exteroceptive, and is the hub for monitoring and controlling bodily states, their integration into consciousness, and emotional and social behavior [36]. The close interaction between sensorimotor and interoceptive information in the insula is exemplified by the modulation of healthy individuals’ postural control during imagined deep pain [37], the altered body schema observed in patients with modified interoception [38], and by the role of interoception in improving postural balance in multiple sclerosis [39]. Also, similar neural correlations have been observed during physical activity and interoceptive tasks [40], which indicates the reciprocal modulation of interoception and motor activity [41,42,43,44,45]. Indeed, exercise interventions improve both interoceptive sensibility and mental health [46].

### 1.3. Aim of the Study

Given the relation of physical activity with motor imagery [18], interoception [41,42,43,44,45], and performance satisfaction [10,11], the study investigated (a) the difference in IA and IS between young sedentary females (Control group, C) and age-matched females who have been practicing artistic gymnastics for a few years (Experimental group, E), and (b) the effects of MI training on IA, IS, and subjective satisfaction with the performance in group E. The choice of artistic gymnastic practicing participants was due to the great role that interoception can exert in these people owing to the relation between interoception and motor actions [47,48].

## 2. Materials and Methods

### 2.1. Participants

The study was approved by the Bioethical Committee of the University of Pisa (n.29/2022, 29 July 2022). All participants signed an informed consent.

G power 3.1 statistics indicated a minimum of 52 participants to obtain significant group differences at T0 and session differences in group E (*p* = 0.05 with d = 0.025, and 1 − β = 0.080).

Twenty-seven females who had practiced artistic gymnastics for 10.22 ± 4.89 years (mean ± sd) volunteered for the study and were included in the Experimental group (E, age: (mean ± sd), 16.85 ± 4.55 years). They used to participate in 3 sessions per week, each lasting approximately 2 h.

Twenty-seven age-matched sedentary females were enrolled in the Control group (C, age: (mean ± sd) 17 ± 4.79 years). An anamnestic interview ascertained the absence of physical and mental diseases, attention and sleep disorders, and ongoing pharmacological therapies.

### 2.2. Experimental Procedure

**Figure 1:**
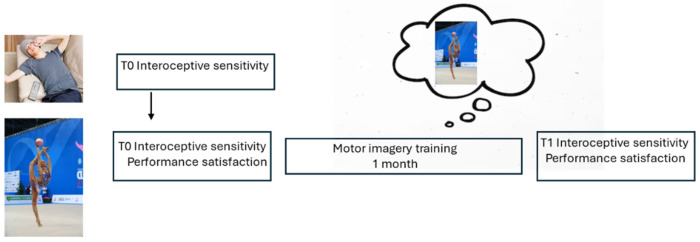
Experimental procedure. Upper level, group C; lower level, group E.

The experimental sessions (Figure 1) were conducted at Jenco Gym (Associazione Sportiva D. Macchianera, Viareggio, Italy) between September and December 2023.

At T0, the participants from both groups completed questionnaires of IS (Multidimensional Assessment of Interoceptive Awareness, MAIA [34]; Body Perception Questionnaire, BPQ [35]; IA (IAS [30], and absorption (Tellegen Absorption Scale, TAS [49]), indicating the ability to focus attention, which is relevant to the efficacy of motor imagery training.

On the same day, E group participants performed their usual sports practice and rated their satisfaction with the performance on a scale ranging from 0 (min) to 10 (max). Then, they were invited to listen to a pre-recorded audio describing their sports activity twice a day in the following month while continuing their sports activity.

At T1, after the training month, group E completed MAIA, BPQ, and IAS questionnaires again, repeated its exercise, and rated satisfaction with the performance (0–10).

### 2.3. Physical Activity

All analyses were performed through the Statistical Package for Social Science (SPSS. 21). After the normality assessment (Kolmogorov–Smirnov), at T0, separate univariate ANOVAs were applied to TAS and IAS, and multivariate ANOVAs were conducted on MAIA and BPQ scores. Groups C and E were the between-subjects factors. The Spearman correlation coefficient between interoception and performance satisfaction was computed in each group.

In group E, T0 and T1 were compared through repeated measure ANOVAs conducted on MAIA, BPQ, IAS, and performance satisfaction.

ANCOVA was used to assess the TAS and previous experience (years of practice), the possible relevance to the changes in interoception (MAIA, BPQ, IAS), and performance satisfaction. The Greenhouse–Geisser correction was applied for non-sphericity. The group E changes (Δ) in interoceptive sensibility, accuracy, and satisfaction with the performance were correlated.

## 3. Results

### 3.1. Group Differences at T0

Separate multivariate analyses revealed significant group differences in TAS (mean ± SD; E (21.96 ± 3.98) > C (18.60 ± 6.05), F(1,53) = 5.72, *p* = 0.020, η^2^ = 0.099, α = 0.651) and MAIA dimensions (F(1,45) = 3.800, η^2^ = 0.354, α = 0.928), and no significant difference in BPQ and IAS (Table 1). The significant MAIA differences consisted of group E higher scores of *attention regulation* (F(1,53) = 11.16, *p* = 0.002), *self-regulation* (F(1,53) = 9.63, *p* = 0.003), *emotional awareness* (F(1,53) = 6.02, *p* = 0.018), *body listening* (F(1,53) = 5.12, *p* = 0.028), and *trusting* (F(1,53) = 4.82, *p* = 0.033).

No significant correlation was observed between trait absorption (TAS) and IAS in either group. After Bonferroni correction, TAS and MAIA dimensions were not significantly correlated.

Only in group E did the number of practicing years positively correlate with MAIA *trusting* (ρ = 0.550, *p* = 0.003) and *quasi*-significantly with *self-regulation* (ρ = 0.374, *p* = 0.055), but the latter did not survive Bonferroni correction (*p* = 0.006).

### 3.2. Group E Differences Between T1 and T0 21.96+

**Figure 2:**
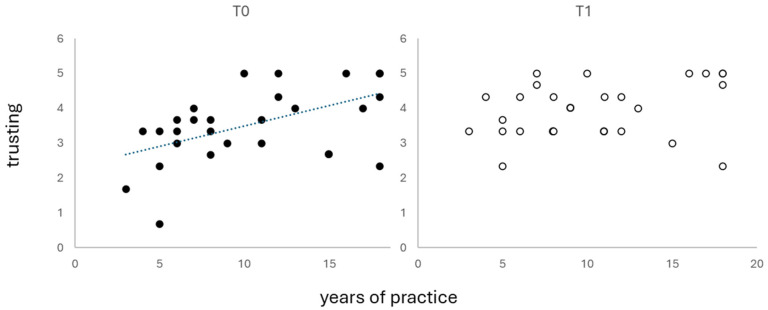
Correlations between practicing years and MAIA *trusting score*.

All participants in group E completed the study. In T1, the correlation between the practicing years and MAIA *trusting* was no longer significant (Figure 2).

**Figure 3:**
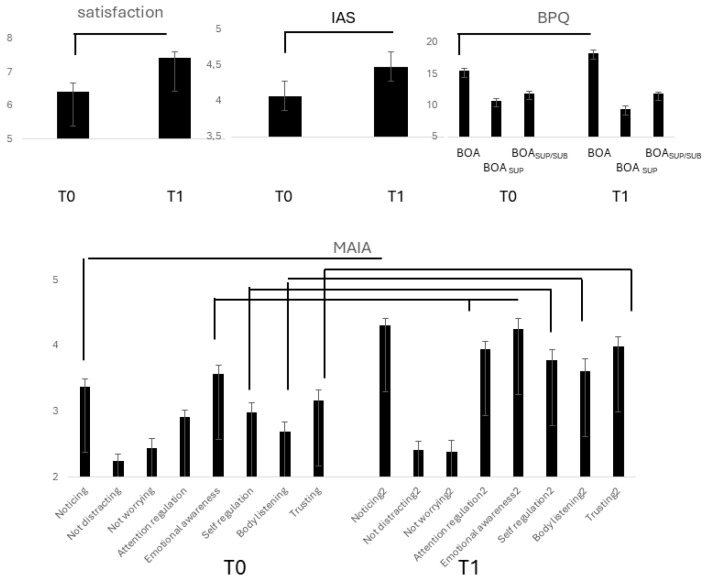
Lines indicate significant differences between T0 and T1 in subjective satisfaction with performance, BPQ, IAS, and MAIA scores in group E.

The level of satisfaction with performance (Figure 3) increased from T0 to T1 (F(1,15) = 35.53, *p* = 0.0001, η^2^ = 0.703, α = 0.999), but the difference was abolished by controlling for the number of practicing years. In T1, it was significantly correlated with MAIA *emotional awareness* (ρ = 0.430, *p* = 0.030). Controlling for the years of sports practice revealed a correlation between performance satisfaction and MAIA *not worrying* (ρ = 0.513, *p* = 0.051).

IAS scores (Figure 3) increased significantly in T1(F(1,26) = 106.07, *p* = 0.0001, η^2^ = 0. 803, α = 0.999), and the difference to T0 remained significant controlling for practicing years and TAS.

BPQ*_BOA_* (Figure 3) increased from T0 to T1 (F(1,26) = 28.11, *p* = 0.0001, η^2^ = 0.520, α = 0.999). Controlling for TAS (*p* = 0.034) and the years of practice maintained significant. BOA*_SUP_* decreased from T0 to T1 (F(1,26) = 21.40, *p* = 0.0001, η^2^ = 0.451, α = 0.991). Controlling for TAS and years of practice abolished the difference.

Separate repeated measure ANOVAs (Figure 3) revealed a significant increase from T0 to T1 in MAIA *noticing* (d.f. 26; F = 73.43, η^2^ = 0.738, α = 0.999), *attention regulation* (F = 68.85, *p* = 0.0001, η^2^ = 0.726, α = 0.999), *emotional awareness* (F = 154.85, *p* = 0.001, η^2^ = 0.364, α = 0.992), *self-regulation*, (F = 11.44, *p* = 0.002, η^2^ = 0.305, α = 0.902), *body listening* (F = 29.61, *p* = 0.0001, η^2^ = 0.552, α = 0.999), *trusting* (F = 13.79, *p* = 0.001, η^2^ = 0.347, α = 0.947). All differences remained unmodified when controlling for the years of practice but became non-significant when controlling for TAS.

The increase in MAIA body listening (Δ) positively correlated with the increases (Δ) in performance satisfaction (ρ = 0.391, *p* = 0.043). Controlling for years of practice disclosed a correlation with not worrying (ρ = 0.434, *p* = 0.027). Nonetheless, no correlation survived Bonferroni correction.

## 4. Discussion

The study provided novel information about the interoceptive differences between sedentary and artistic gymnastics-practicing healthy females and the effects of a 1-month motor imagery training in the latter group.

According to MAIA results, the study indicated that artistic gymnastics-practicing healthy young females exhibit better interoceptive sensibility than sedentary age-matched girls regarding the greater tendency to listen to and trust in the body and to modify attention and emotion according to body sensations, in line with other authors [50]. The better interoceptive sensibility of group E can be attributed to the years of practice, as physical activity enhances interoceptive sensibility [40,50], which indicates that sports practice promotes an adaptive interpretation of bodily signals. Since we did not observe group differences in BPQ dimensions, whose scales are associated with the interoceptive signals from specific body parts, we argue that BPQ information did not contribute to the MAIA-reported baseline interoceptive sensibility.

Interoceptive accuracy (IAS) did not differ between the groups, at variance with earlier reports of better interoceptive accuracy in sports-practicing individuals compared to [51] and in athletes [50] compared to controls. Inconsistent results could be attributed to the participants’ different physical levels [50].

The dissociation between interoceptive accuracy and sensibility can be accounted for by their different functional meanings [52] and has been observed in healthy individuals [53] and various clinical conditions [27].

Trait absorption did not influence interoceptive sensibility (MAIA, BPQ) and accuracy (IAS), in line with the scarce relevance of attentional abilities to interoceptive accuracy measured by heartbeats counting [40,45,54].

### 4.1. Differences Between T1 and T0 in Group E

Motor imagery training improved interoceptive accuracy, sensibility, and satisfaction with performance from T0 to T1. We cannot state whether the increase in performance satisfaction was due to performance improvement, as this could not be measured. Since the increase in satisfaction became non-significant only controlling for the years of practice, the imagery training did not influence it after training owing to practice-related ceiling effects.

At variance with practice, the ability to focus attention (TAS) was involved in the efficacy of motor training, as the difference between T1 and T0 in interoceptive sensibility indicated by MAIA became non-significant when controlling for TAS scores. In contrast, the increase in BPQ_*BOA*_ score was independent of absorption and earlier practice, suggesting the role of autonomic activity in the observed improvement in interoceptive sensibility. Of interest, motor training per se would reduce the parasympathetic activity of the upper body part (BOA_*SUP*_), but absorption and earlier practice mask such an effect.

The improvement in interoceptive sensibility suggests greater ease in motor activity, as its increase has been associated with greater body confidence, attention, self-esteem, and better self-regulation [55], which could account for greater performance satisfaction.

The increase in self-reported interoceptive accuracy (IAS), independent from both earlier practice and trait absorption, accords with reports describing its increase after 2 weeks of physical training without motor imagery [51]. Thus, interoceptive accuracy seems more closely related than sensibility to actual motor activity.

The changes in the association between self-reported satisfaction and MAIA dimensions observed after training, when they were no longer correlated, suggest that the differences in satisfaction might be due to increased interoceptive sensibility enhancing the relevance of bodily signals to the construction of consciousness. This would agree with the observation that interoceptive sensibility [24]) and accuracy ([24]) are involved in the cortical correlations of motor imagery and are based on the shared elaboration of interoceptive and exteroceptive signals by the insula [36]. Their integration is highlighted by studies reporting better tactile sensibility during diastole rather than systole and a greater likelihood of voluntary actions starting during insertion rather than exhalation, as well as by the suppression of EEG differences during imagery by the influence of interoceptive sensitivity [24] and accuracy ([24]).

### 4.2. Limitations and Conclusions

A limitation of the study is that we could not recruit a control group performing the same sport not submitted to motor training. Other limitations are the absence of an assessment of motor imagery cortical correlations, which prevented the evaluation of its efficacy by objective methods and of objective measures of interoceptive accuracy, i.e., heartbeat counting and heartbeat-evoked cortical potentials. These variables could not be acquired in the gym. Moreover, present findings cannot be extended to the general population, as only females were enrolled in the study, and to different sports that could be influenced by motor imagery differentially.

In conclusion, the baseline differences between the two studied groups confirm earlier reports of better interoceptive sensibility in sports practicing people [46]. The comparison between pre- and post-training sessions in the artistic gymnastics-practicing group shows that previous sports experience is associated with further improvement in interoceptive accuracy and sensibility after motor imagery training, in line with the intermingled pathways and shared relay stations of sensorimotor and interoceptive information [56]. Attentional abilities likely inducing better motor imagery account for the improvement in interoceptive sensibility but not for the increased interoceptive accuracy.

The findings are relevant to medicine in the perspective of body–mind treatments applied to patients with low interoceptive sensitivity and/or accuracy—like persons with depersonalization/derealization or eating disorders, respectively [27]—that could improve their interoception by actual and imagined physical activity.

## Figures and Tables

**Table 1:** Interoception and performance satisfaction at T0 and T1 (mean, standard deviation).

		Group C		Group E			
Questionnaire	Scale	T0		T0		T1	
MAIA		mean	SD	mean	SD	mean	SD
	*Noticing*	3.18	0.93	3. 57	0.82	4.31	0.56
	*Not distracting*	2.10	0.70	2.38	0.85	2.42	0.65
	*Not worrying*	2.46	1.16	2.43	0.93	2.38	0.93
	*Attention regulation* *	2.61	0.58	3.23	0.77	3.94	0.62
	*Emotional awareness* *	2.56	1.22	3.87	0.74	4.25	0.82
	*Self-regulation* *	2.57	1.22	3.42	0.74	3.79	0.78
	*Body listening* *	2.38	1.04	3.01	1.01	3.62	0.93
	*Trusting* *	2.82	1.27	3.52	1.07	3.99	0.81
BPQ	*BOA*	15.00	2.87	15.93	3.23	18.30	2.2
	*SUP*	10.81	2.92	10.67	2.42	9.48	1.97
	*BOASUP*	12.00	2.75	11.78	2.5	11.85	1.63
IAS *		3.87	0.70	4.48	0.55	6.59	0.38
Performance	satisfaction *			6.59	0.95	7.44	0.79

Note. * significant group differences at T0.

## Data Availability

Data will be available upon request.
